# Myomegalin is a novel A-kinase anchoring protein involved in the phosphorylation of cardiac myosin binding protein C

**DOI:** 10.1186/1471-2121-12-18

**Published:** 2011-05-10

**Authors:** Gerrida M Uys, Amsha Ramburan, Benjamin Loos, Craig J Kinnear, Lundi J Korkie, Jomien Mouton, Johann Riedemann, Johanna C Moolman-Smook

**Affiliations:** 1 US/MRC Centre for Molecular and Cellular Biology, Department of Biomedical Sciences, University of Stellenbosch, South Africa; 2 Central Analytical Facility, Department of Physiology, University of Stellenbosch, South Africa

## Abstract

**Background:**

Cardiac contractility is regulated by dynamic phosphorylation of sarcomeric proteins by kinases such as cAMP-activated protein kinase A (PKA). Efficient phosphorylation requires that PKA be anchored close to its targets by A-kinase anchoring proteins (AKAPs). Cardiac Myosin Binding Protein-C (cMyBPC) and cardiac troponin I (cTNI) are hypertrophic cardiomyopathy (HCM)-causing sarcomeric proteins which regulate contractility in response to PKA phosphorylation.

**Results:**

During a yeast 2-hybrid (Y2H) library screen using a trisphosphorylation mimic of the C1-C2 region of cMyBPC, we identified isoform 4 of myomegalin (MMGL) as an interactor of this N-terminal cMyBPC region. As MMGL has previously been shown to interact with phosphodiesterase 4D, we speculated that it may be a PKA-anchoring protein (AKAP).

To investigate this possibility, we assessed the ability of MMGL isoform 4 to interact with PKA regulatory subunits R1A and R2A using Y2H-based direct protein-protein interaction assays. Additionally, to further elucidate the function of MMGL, we used it as bait to screen a cardiac cDNA library. Other PKA targets, viz. CARP, COMMD4, ENO1, ENO3 and cTNI were identified as putative interactors, with cTNI being the most frequent interactor.

We further assessed and confirmed these interactions by fluorescent 3D-co-localization in differentiated H9C2 cells as well as by *in vivo *co-immunoprecipitation. We also showed that quantitatively more interaction occurs between MMGL and cTNI under β-adrenergic stress. Moreover, siRNA-mediated knockdown of MMGL leads to reduction of cMyBPC levels under conditions of adrenergic stress, indicating that MMGL-assisted phosphorylation is requisite for protection of cMyBPC against proteolytic cleavage.

**Conclusions:**

This study ascribes a novel function to MMGL isoform 4: it meets all criteria for classification as an AKAP, and we show that is involved in the phosphorylation of cMyBPC as well as cTNI, hence MMGL is an important regulator of cardiac contractility. This has further implications for understanding the patho-aetiology of HCM-causing mutations in the genes encoding cMyBPC and cTNI, and raises the question of whether MMGL might itself be considered a candidate HCM-causing or modifying factor.

## Background

Cardiac contractility is significantly enhanced by the dynamic phosphorylation of a number of sarcomeric proteins, including cardiac myosin binding protein C (cMyBPC) [1,2]. This highly modular protein, found in the C-zone of the sarcomere, is encoded by a gene which is frequently implicated in hypertrophic cardiomyopathy (HCM) [3], a common inherited cardiac disease characterised by hypertrophy of the ventricular muscle [4]. There are multiple isoforms of this protein; the cardiac isoform differs from its skeletal counterparts by containing an extra immunoglobulin-like (IgI) domain (C0) at the amino terminal, a charged residue-rich insertion in domain C5 and three phosphorylation sites in a motif between the second and third IgI domains (C1-C2), known as the MyBPC motif or m-domain. Originally thought to have only a structural role, cMyBPC has been shown to play an important role in the regulation of cardiac contractility [1], for which the N-terminal region of the protein appears to be crucial. Upon β-adrenergic stimulation, three sites within the MyBPC motif are phosphorylated by protein kinase A (PKA) and calcium/calmodulin-activated protein kinase (CaMK), the phosphorylation of this region of MyBPC then leads to rearrangement of the myosin crossbridges and thick filament structure [1,5,6] such that cardiac contractility is increased [7].

However, since these kinases regulate a broad range of cellular responses, their compartmentalization in close proximity to their sarcomeric targets is required to facilitate control over which proteins are phosphorylated in response to second messenger signalling [8,9]. At the same time, co-compartmentalization of enzymes or proteins that generate or terminate these second messenger metabolites, such as the phosphodiesterases (PDEs) which degrade cAMP and cGMP, with the relevant responsive kinases helps to optimise the precision and speed of response to second messenger signaling [10]. Compartmentalization of kinases in general is achieved by either direct docking of the kinase on the target protein, or by anchoring of the kinase to, or close to, the target via an adaptor protein, named A-kinase anchoring proteins or AKAPs in the case of PKA [11]. Although a CaMK has been found to co-purify with cMyBPC [1,12], the mechanism of co-compartmentalization of cMyBPC and PKA has never been described and very little is known about sarcomeric AKAPs in general.

In this study we identified myomegalin (MMGL) isoform 4, a PDE4D-interacting protein [13], as a binding partner of PKA, the cMyBPC N-terminal region, as well as other PKA-targets, and show that MMGL meets all the requirements for classification as a novel sarcomeric AKAP, with important implications for regulation of cardiac contractility during adrenergic stimulation.

## Results

### Interaction of trisphospho-cMyBPC with MMGL

As the interactions of the N-terminal region of cMyBPC under its various phosphorylation states appear to be integral to the regulatory role of cMyBPC in contractility, we aimed to gain further insight into the interactions of the trisphosphorylated form of the N-terminal region of cMyBPC. Thus, we used a construct of C1-C2 in which the serines in the three phosphorylation sites of the MyBPC motif had been replaced by glutamic acids (PPP), mimicking the trisphosphorylated state, as bait in a Y2H cardiac library screen.

During successive rounds of nutritional and colorimetric selection, 19 putative interactors of PPP were identified, which were able to activate the *HIS3, ADE2 *and *MEL1 *reporter genes in the presence of the PPP bait, but not in the presence of heterologous baits (Table 1). Of these, three in-frame prey plasmids encoded isoform 4 of PDE4D-interacting protein, also known as myomegalin (MMGL) (Table 1). This putative interaction between MMGL and the N-terminal of cMyBPC was intriguing, as the former protein is known to anchor PDE4D to particulate structures; PDE4D, in turn, is known to hydrolyze cAMP and thus to attenuate PKA-driven phosphorylation [13]. Furthermore, it has been shown that some adaptor proteins can anchor both PKA and PDE4D [14], raising the possibility that MMGL may be anchoring PKA to at least cMyBPC in the sarcomere as an AKAP; hence this interaction was prioritized for further investigation.

**Table 1:** Interactors of the C1-C2 region of cMyBPC in the trisphospho-mimic state as identified by yeast 2-hybrid library screening

Clone #	Genomic Hit, Accession no (E-value)	In-frame ORF Protein Hit, Accession no (E-value)	Subcellular localization
P205, P272, P403	PDE4DIP, transcript 4 NM_001002810 (0.0)	*Homo sapiens *phosphodiesterase 4D interacting protein (myomegalin) NP_055459.3 (1e-70)	Cytoplasm, Cytoskeleton
P182, P286, P267	ACTC1 NM_005159 (0.0)	*Homo sapiens *actin, alpha, cardiac muscle 1 NP_005150 (1e-89)	Thin filament sarcomere
P259	ACTCB NM_001101 (0.0)	*Homo sapiens *actin, beta AAH23548.1 (8e-34)	Cytoplasm, cytoskeleton
P62, P213, P319	MYBPC3 NM_000256 (0.0)	*Homo sapiens *cardiac myosin binding protein C, NP_000247.2 (2e-26)	Thick filament; Sarcomere
P2,P81	Gcom1 transcript 12 NM_001018100 (0.0)	*Homo sapiens *GRINL1A combined protein NP_001018100.1, (2e-65)	Unknown
P80	FLNC NM_001458.2 (0.0)	*Homo sapiens *filamin C, gamma NP_001449.3 (2e-91)	Myofibrillar z-disks; actin cytoskeleton
P175, P465	CNN1 NM_001299.4 (0.0)	*Homo sapiens *calponin 1, basic, smooth muscle NP_001290.2 (1e-104)	Cytoplasm, cytoskeleton
P11	PARD3 NM_019619.2 (3e-121)	*Homo sapiens *par-3 partitioning defective 3 homolog NP_062565.2 (1e-24)	Plasma membrane; tight junctions
P223	CCT7 NM_001009570.1 (0.0)	*Homo sapiens *chaperonin containing TCP1, subunit 7 (eta) NP_001009570.1 (1e-83)	Cytoplasm
P238	SVIL NM_021738.1 (0.0)	*Homo sapiens *supervillin NP_003165.1 (1e-15)	Actin cytoskeleton, plasma membrane, nucleus
P398	FLJ21347 NM_022827.2 (0.0)	FLJ21347 NP_073738.2 (4e-101)	Unknown

**Figure 1:**
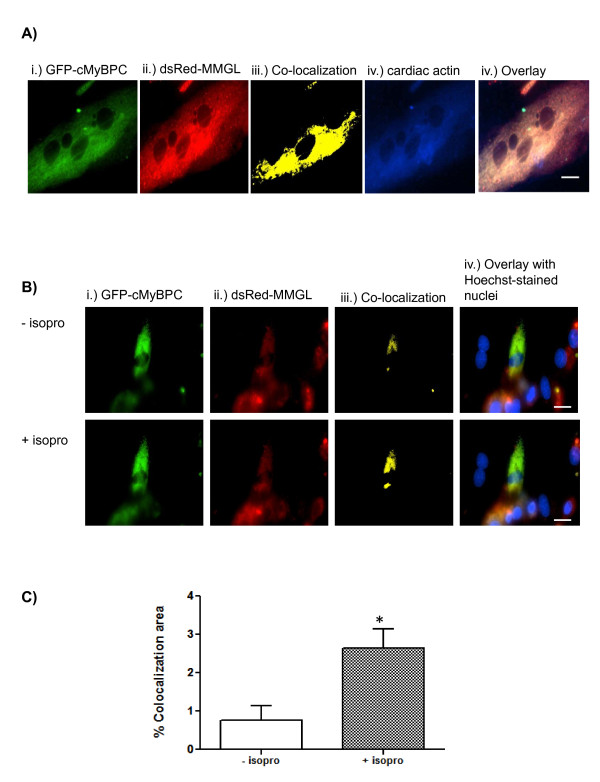
**MMGL isoform 4 interacts with the C1-C2 region of cMyBPC**. **A**. Representative image of live cell fluorescence microscopy showing co-localization of cMyBPC and MMGL isoform 4. Each panel represents a single frame of the 25 images that were captured for the vertical Z-stack. The first four panels shows a single colour channel, while the image in the last panel shows an overlay of the four colour channels used. Column (iii) shows co-localization (yellow fluorescence) between dsRed-MMGL and GFP-cMyBPC, while column (iv) shows cardiac actin, a marker of the sarcomeric region. Scale bar: 0.02 mm. **B**. Representative image of live cell fluorescence microscopy showing that co-localization of MMGL isoform 4 and cMyBPC increases under adrenergic stress. Each panel represents a single frame of the 25 images that were captured for the vertical Z-stack. Each of the first three columns shows a single colour channel, while the image in the last column shows an overlay of the four colour channels used. Column (iii) shows co-localization (yellow fluorescence) between dsRed-MMGL and GFP-cMyBPC in the absence (-isopro) and presence (+isopro) of the beta-adrenergic agonist, isoproterenol. As evidenced by the increased yellow staining, co-localization levels between MMGL and cMyBPC increased ten minutes after the addition of isoproterenol. Scale bar: 0.02 mm. **C**. Quantification of co-localization shown in B demonstrates the significant increase in co-localization after the addition of isoproterenol (± SEM, *p < 0.05, *n *= 5). Change in co-localization was calculated using the CellR software and presented as a false colour image and percent co-localization as described by Loos *et al*., 2008 [29]. *Abbreviations: *isopro = isoproterenol

3D-fluorescence microscopy indicated that cMyBPC and MMGL isoform 4 colocalize in the cytoplasmic (encompassing the sarcomeric) region of differentiated rat cardiac H9C2 cells (Figure 1A). Exposure of the cells to the adrenergic agonist isoproterenol led to an increase in co-localization of the two proteins, as evidenced by the increased yellow staining during fluorescence microscopy of such cells (Figure 1B, C).

**Figure 2:**
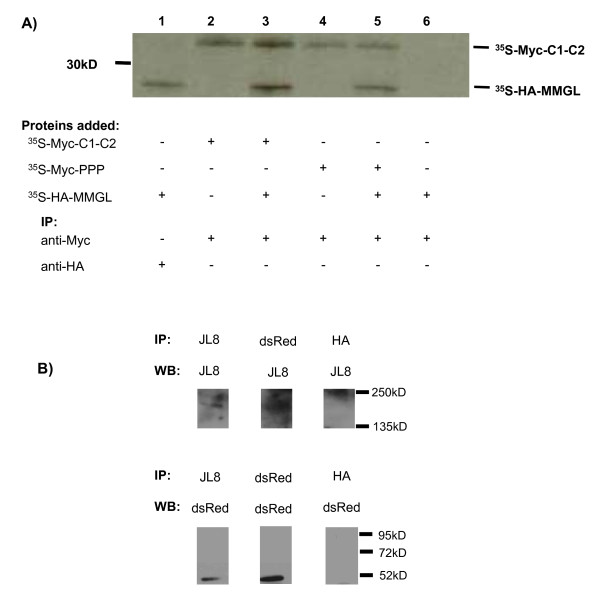
**Co-immunoprecipitations of MMGL isoform 4 and the C1-C2 region of cMyBPC**. **A**. *In vitro *co-immunoprecipitation showing that MMGL interacts with the native as well as the trisphospho-mimic of C1-C2 in the absence of Y2H GAL4 domains. **B**. *In vivo *co-immunoprecipitation, showing that dsRed-tagged MMGL is able to specifically pull down GFP-cMyBPC, and *vice versa*, in H9C2 cells. *Abbreviations: *JL8 = antibody directed against GFP, dsRed = antibody directed against dsRed-tagged proteins, HA = antibody directed against haemaglutinin protein, used as negative control antibody, IP = antibody used in immunoprecipitation, WB = antibody used in western blotting.

Furthermore, in order to verify that it is the C1-C2 region of cMyBPC that interacts with MMGL isoform 4, specifically, and does so in the absence of the GAL4 regions of the Y2H bait protein, we used *in vitro *co-immunoprecipitation assays. We also used these assays to assess whether trisphosphorylation of the MyBPC motif within this region was crucial to the putative interaction. Both the native C1-C2 and a trisphospho-mimic of the C1-C2 region interacted with MMGL isoform 4 in the absence of the Y2H GAL4 domains (Figure 2A), suggesting that the interaction can take place whether or not the MyBPC-motif is phosphorylated. Interaction between cMyBPC and MMGL isoform 4 was further confirmed in a cellular environment by reciprocal co-immunoprecipitation of these tagged proteins in H9C2 cells (Figure 2B).

### MMGL binds to PKA regulatory subunits

**Figure 3:**
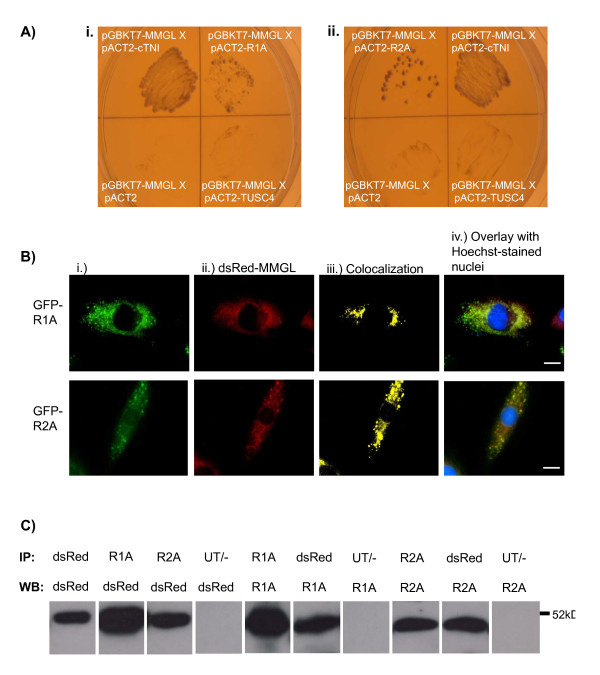
**MMGL acts as a dual-specific AKAP by anchoring PKA regulatory isoforms R1A and R2A**. **A**. Activation of nutritional reporter genes by PKA-MMGL interaction during direct protein-protein interaction assays. Small-scale Y2H matings between pGBKT7-MMGL and pACT2-PRKAR1A (i) and pACT2-PRKAR2A (ii) on solid medium lacking Leu, Trp, His and Ade. Growth of yeast yells indicate interaction of MMGL with PRKAR1A as well as PRKAR2A. **B**. Representative images of live cell fluorescence microscopy showing co-localization of MMGL with PRKAR1A and PRKAR2A in differentiated H9C2 cardiomyocytes. (i) GFP-tagged PRKAR1A and PRKAR2A is seen in the cytosol as green fluorescence. (ii) dsRed-tagged MMGL expression is seen as red fluorescence. (iii) Yellow fluorescence indicates the co-localization of PRKAR1A and PRKAR2A with MMGL. (iv) Overlay of images A-C with Hoechst H-33342 labelling of the nuclei (blue fluorescence), for orientation purposes. Scale bar: 0.02 mm. **C**. Western blots of *in vivo *co-immunoprecipitations of PKA regulatory subunits and MMGL; the antibodies used in immunoprecipitation (IP) and Western Blot (WB) are shown above each lane. Endogenous PRKAR1A and PRKAR2A immunoprecipitated exogenous dsRed-MMGL in lysates of dsRed-MMGL-transfected, differentiated H9C2 cardiomyocytes. Conversely, PRKAR1A and PRKAR2A also immunoprecipitated exogenous dsRed-MMGL in these lysates. The negative control lanes included lysates from cells not transfected with dsRed-MMGL, showing that these precipitations are not spurious, but are the result of physical association between the relevant proteins. *Abbreviations: *Prot G = protein G control; R1A = PRKAR1A; R2A = PRKAR2A, UT/- = negative control lanes.

As the primary characteristic of an AKAP is the ability to bind at least one PKA regulatory subunit, we investigated the possibility that MMGL functions as an AKAP by using Y2H-based direct protein-protein interaction assays to determine binding between MMGL and the two cardiac-expressed regulatory subunits of PKA. Both regulatory subunits (PRKAR1A and PRKAR2A) interacted with MMGL, as assessed by growth and light colour of the colonies on quadruple dropout nutritional selection medium, compared to relevant controls (Figure 3A).

Further support for this finding was garnered by co-localization studies using 3D-fluorescence microscopy: we demonstrated that MMGL isoform 4 occurs in the same 3D-subcellular region as the two PKA regulatory isoforms in H9C2 cardiomyocytes (Figure 3B).

To further verify physical interaction between MMGL and these PKA isoforms in a cellular context and in the absence of GAL4 domains, we performed pull-down assays in differentiated H9C2 cardiomyocytes. As commercial antibodies against MMGL/PDE4DIP are not able to detect isoform 4, the smallest isoform of this protein, we used an antibody directed against dsRed to detect a dsRed-tagged version of MMGL isoform 4 in these assays. In this way, endogenous PRKAR1A and PRKAR2A were shown to immunoprecipitate dsRed-MMGL, and *vice versa *(Figure 3C), indicating physical interaction of MMGL with these two PKA regulatory isoforms.

### MMGL binds to additional PKA targets

We further investigated the function of MMGL isoform 4 by using it as Y2H bait to screen a cardiac cDNA library in order to identify its additional binding partners. Thirteen in-frame putative MMGL-interactors were identified that activated all three nutritional reporter genes in the presence of the MMGL bait, but not in the presence of heterologous baits (Table 2). As we were primarily interested in the possibility of MMGL acting as a sarcomeric AKAP, proteins with defined vesicular localizations were not considered of primary interest for follow-up in this study; these included the mitochondrial protein COX5A, the proteosome 26S subunit and the endosomal protein SNX3. Of the remaining ten putative MMGL interactors, six encoded cardiac troponin I (cTNI) (Table 2).

**Table 2:** Interactors of MMGL isoform 4 identified by yeast 2-hybrid library screening

Clone #	Genomic Hit, Accession no & E-value	In-frame ORF Protein Hit, Accession no & E-value	Subcellular localization
128, 137, 148, 149, 160, 200	TNNI3 NM 000363, 0.0	*Homo sapiens *troponin I type 3 (cardiac) NP 000354.3, 2e-82	Thin filament
192	CARP NM 014391, 0.0	*Homo sapiens *Cardiac ankyrin repeat protein NP 055206, 2e-121	Cytosol, nucleus
715	COMMD4 NM 017828, 0.0	*Homo sapiens *COMM domain containing 4 NP 060298.2, 3e-97	Cytoplasm
130	ENO1 NM 001428, 0.0	*Homo sapiens *Enolase 1 (alpha) NP 001419, 4e-116	Cytoplasm
203	ENO3 NM 053013, 0.0	*Homo sapiens *Enolase 3 (beta, muscle) NP 001967.1, 000.1	Cytoplasm

**Figure 4:**
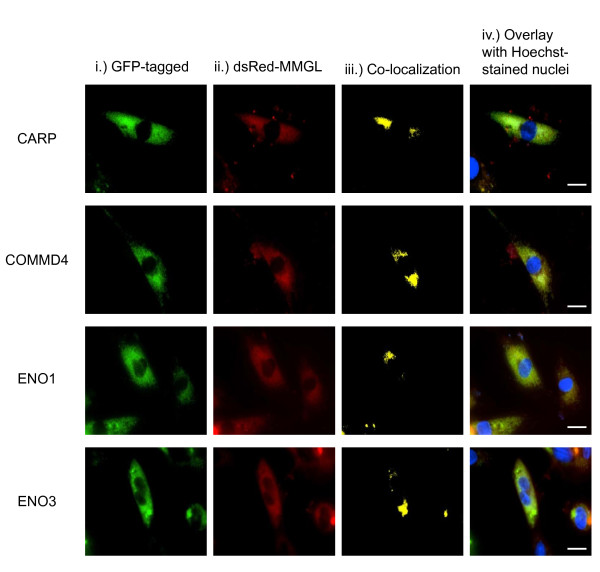
**3D co-localization of MMGL and its respective preys identified in the Y2H library screen**. Representative images of live cell fluorescence microscopy showing co-localization of MMGL and the putative interactors identified in the Y2H library screen in differentiated H9C2 cardiac myocytes. (i) Individual GFP-tagged putative library screen interactors are seen as green fluorescence, as indicated by labels to the left of the row. (ii) dsRed-tagged MMGL expression in the same cell(s) are shown are red fluorescence. (iii) Co-localization of interactors and MMGL within these cell(s), generated from 3D vertical Z-stack images, are shown as yellow fluorescence. (iv) Overlay of images A-C with Hoechst H-33342 labelling of the nuclei (blue) for orientation purposes. The presence of yellow staining in each of the images in (iii) indicates that each of the respective preys colocalize with MMGL in differentiated H9C2 cardiomyocytes. Scale bar: 0.02 mm

**Figure 5:**
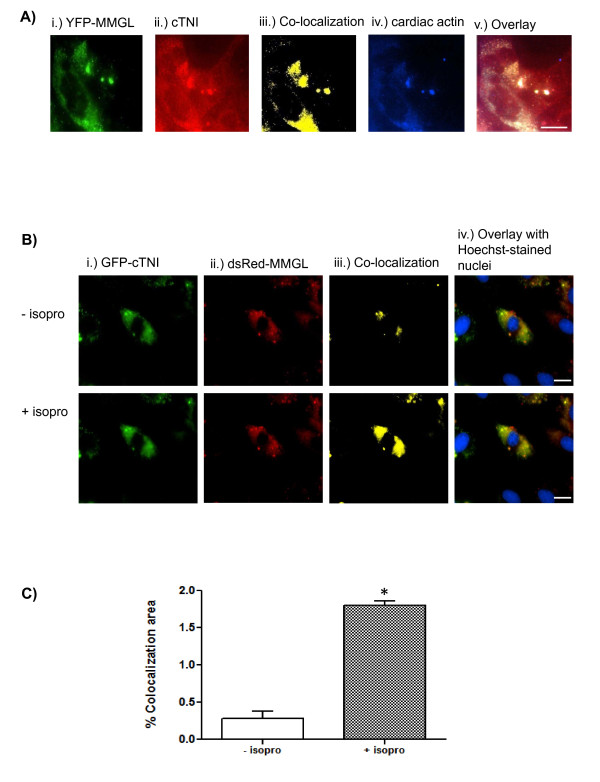
**Co-localization increases between MMGL and PKA target upon β-adrenergic stimulation**. **A**. Representative image of live cell fluorescence microscopy showing co-localization of cTNI and MMGL isoform 4. Each panel represents a single frame of the 25 images that were captured for the vertical Z-stack. The first four panels show a single colour channel, while the image in the last panel shows an overlay of the four colour channels used. Column (iii) shows co-localization (yellow fluorescence) between dsRed-cTNI and YFP-MMGL, while column (iv) shows cardiac actin, a marker of the sarcomeric region. Scale bar: 0.02 mm. **B**. Representative image of live cell fluorescence microscopy showing that co-localization of MMGL isoform 4 and cTNI increases under adrenergic stress. Each panel represents a single frame of the 25 images that were captured for the vertical Z-stack. Each of the first three columns shows a single colour channel, while the image in the last column shows an overlay of the four colour channels used. Column iii shows co-localization (yellow fluorescence) between dsRed-MMGL and GFP-cTNI in the absence (-isopro) and presence (+isopro) of the beta-adrenergic agonist, isoproterenol. The increase in intensity of yellow fluorescence in the second row demonstrates that co-localization levels of MMGL and cTNI had increased ten minutes after the addition of isoproterenol. Scale bar: 0.02 mm. **C**. Quantification of co-localization shown in B shows that co-localization increased significantly (± SEM, *p < 0.05, *n *= 6) after the addition of isoproterenol. Change in co-localization was calculated using the CellR software and presented as a false colour image and percent co-localization as described by Loos *et al*., 2008 [29]. *Abbreviations: *isopro = isoproterenol

Further support for the validity of these interactions was provided by finding that MMGL occurs in the same 3D-subcellular region as all five of the putative interactors identified in the Y2H library screen, viz. cardiac ankyrin repeat protein (CARP), copper metabolism gene *MURR1 *domain 4 (COMMD4), α-enolase (ENO1), β-enolase (ENO3) (Figure 4), and cardiac troponin I (cTNI) (Figure 5), in differentiated cardiomyocytes.

**Figure 6:**
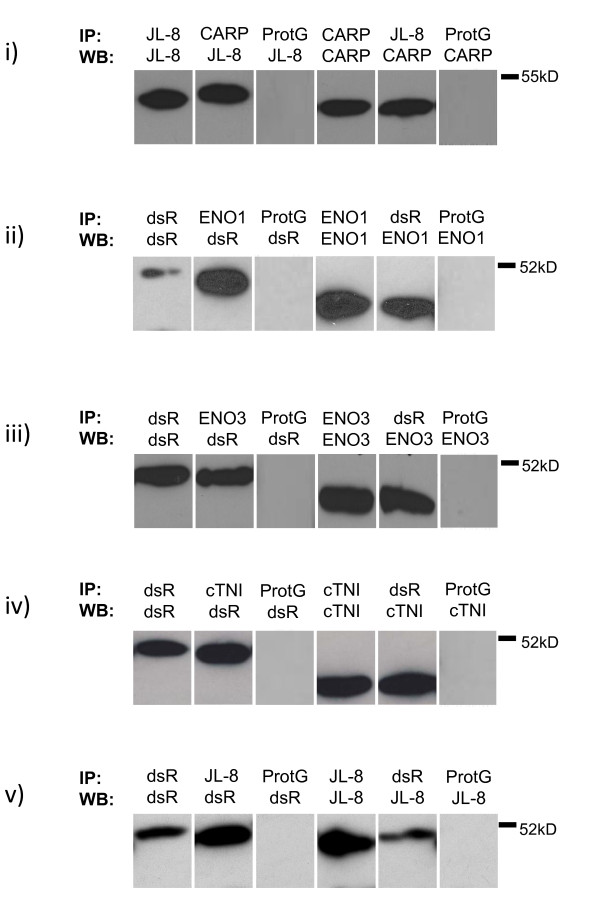
***In vivo *co-immunoprecipitation of MMGL and its respective preys identified in the Y2H library screen**. Western blots supporting the co-localization data in Figures 4 and 5. Antibodies used in immunoprecipitation (IP) and Western Blot (WB) are shown above each lane. Endogenous CARP (i), ENO1 (ii), ENO3 (iii) and cTNI (iv) immunoprecipitated exogenous dsRed-/YFP-MMGL *in vivo *in lysates of ds-Red-/YFP-MMGL transfected, differentiated H9C2 cardiomyocytes, while GFP-COMMD4 immunoprecipitated dsRed-MMGL in differentiated H9C2 cardiomyocytes transfected with both GFP-COMMD4 and dsRed-MMGL. Conversely, dsRed- or YFP-MMGL immunoprecipitated endogenous CARP (i), ENO1 (ii), ENO3 (iii) and cTNI (iv) in lysates of ds-Red- or YFP-MMGL transfected, differentiated H9C2 cardiomyocytes, while dsRed-MMGL also immunoprecipitated GFP-COMMD4. The dsRed antibody is directed against the dsRed-MMGL fusion protein, while the JL-8 antibody is directed against the YFP/GFP fusion proteins. The clear protein G control lanes show that these precipitations are not spurious, but are the result of physical association between the relevant proteins. Similar clear lanes were obtained when the HA antibody was used in negative control immunoprecipitation reactions (data not shown). *Abbreviations: *Prot G = protein G control; JL8 = antibody directed against YFP-tagged proteins, dsR = antibody directed against dsRed-tagged proteins, as described above.

Moreover, in pull-down assays, exogenous, fluorescently-tagged MMGL and endogenous ENO1, ENO3, CARP and cTNI reciprocally co-precipitated each other (Figure 6i-iv). As COMMD4 had a similar mobility to antibody light chains, which interfered with detection of these proteins in Western blots, a GFP-tagged fusion of this protein was expressed in H9C2 cells for pull-down assays. In these assays, exogenous GFP-COMMD4 immunoprecipitated exogenous dsRed-MMGL, and *vice versa *(Figure 6v). Thus, Western blot analysis data supported the proposed interaction of MMGL with ENO1, ENO3, CARP, cTNI and COMMD4.

cTNI is a known PKA target [15], while the remaining four putative interactors were shown to be likely targets using Phosphomotif Finder http://www.hprd.org/PhosphoMotif_finder; we therefore investigated the effect of isoproterenol stimulation of the H9C2 cells on co-localization, using the most frequent, and sarcomeric-located, putative interactor, cTNI, as example. Treating H9C2 cells transfected with dsRed-MMGL/GFP-cTNI with isoproterenol resulted in significantly more 3D co-localization between these two proteins, as shown in Figure 5B and 5C, compared to non-treated cells.

Thus, our results strongly suggest that MMGL interacts with both PKA regulatory isoforms, as well as with each of the prioritized five putative prey interactors identified in the Y2H screen, in a cellular milieu, and in the absence of the GAL4 domains.

### Effect of MMGL knockdown

**Figure 7:**
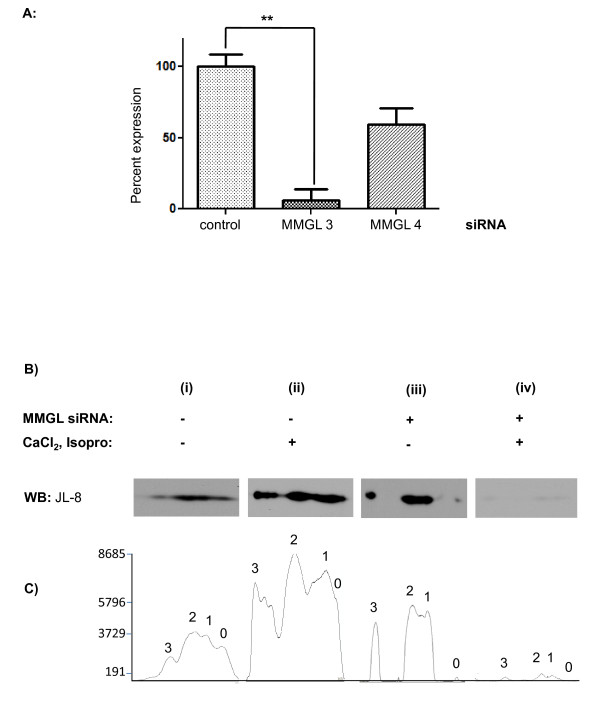
**siRNA-mediated knockdown of MMGL leads to reduced levels of cMyBPC phosphorylation under adrenergic stimulation**. **A**. Graph demonstrating that real-time quantification of *MMGL *cDNA transcribed from RNA extracted from cells transfected with either a non-silencing control or a particular *MMGL *siRNA. *Pde4dip *Rn_RGD:708410_3_HP (MMGL 3) resulted in optimal knockdown of *MMGL *(80%). **B**. Western blots of 2-dimensional IEF gels showing the expression of the four phosphorylation isoforms of GFP-cMyBPC in H9C2 cells (i) under non-stimulated conditions; (ii) under adrenergic stimulation, (iii) under non-stimulated conditions in the absence of MMGL (i.e. with MMGL knock-down) and (iv) under adrenergic stimulation in the absence of MMGL. **C**. Quantification of cMyBPC isoforms in the autoradiographs of the 2-dimensional IEF gels shown in (B), graphing the levels of the four phosphorylation isoforms (0 = unphosphorylated; 1 = monophosphorylated, 2 = diphosphorylated, 3 = trisphosphorylated). B and C(i) show that under non-stimulated conditions, levels of the mono- and diphosphorylated forms are similar and increased compared to the trisphosphorylated form. B and C(ii) show that under adrenergic stimulation, there is a relative increase in the trisphosphorylated form of cMyBPC and reduction of the non-phosphorylated form. B and C (iii) show that under non-stimulated conditions, ratios of the four forms of cMyBPC are similar to that in wild-type cells. B and C(iv) show that, upon adrenergic stress, knock-down of MMGL leads to a reduction in the levels of all cMyBPC phosphorylation forms. *Abbreviations: *JL8 = antibody directed against GFP-MyBPC.

In order to evaluate the role of MMGL in cMyBPC phosphorylation, we assessed the expression of the different phosphorylation isoforms of cMyBPC, in the context of siRNA-mediated MMGL knockdown. Typically, interference with AKAP-functioning is commonly more noticeable on the target protein only after adrenergic stimulation [16], thus we also tested the effect of MMGL knockdown in the presence of adrenergic stimulation. Of four siRNAs tested, Rn_RGD:708410_3_HP siRNA (MMGL 3) (Qiagen) was found to provide optimal knockdown of MMGL (80%) in H9C2 cells (Figure 7A); thus MMGL 3 siRNA was used in subsequent experiments to silence MMGL gene expression.

Using Western blots of 2-dimensional IEF gels, we found that similar amounts of the mono- and diphosphorylated forms of cMyBPC were expressed in untreated H9C2 cells, while lesser amounts of the un- and trisphosphorylated forms, relative to the other isoforms, were present (Figure 7Bi, Ci). When these cells were exposed to increased CaCl_2 _and isoproterenol, to activate CaMK and PKA such that maximum phosphorylation of the MyBPC-motif is likely[7], the relative amount of the trisphosphorylated form increases markedly, while the relative amount of unphosphorylated cMyBPC decreased (Figure 7Bii, Cii). In untreated cells lacking MMGL, all four forms of cMyBPC are present, with amounts of mono- and diphosphorylated forms similar and exceeding the amounts of un- and trisphosphorylated forms, similar to what was seen with untreated, wild-type cells. However, adrenergic stimulation in the presence of MMGL knockdown resulted in very low expression of all isoforms (Figure 7Biv, Civ). This finding is compatible with previous findings of an inverse relationship between phosphorylation of cMyBPC and its proteolytic degradation, suggesting that phosphorylated cMyBPC is protected against proteolytic cleavage, whereas absence of phosphorylation results in increased degradation of the protein and reduced levels of cMyBPC in the cell [17,18].

## Discussion

Myomegalin has been characterized as a protein with the properties of a scaffold or structural protein that is expressed at high levels in skeletal and cardiac tissue, suggesting an important function in muscle, and which interacts with a cAMP-specific phosphodiesterase [13]. However, the precise function and interactions of this protein, and its five isoforms, have been largely unknown. We here describe how the smallest MMGL isoform, isoform 4, binds to known and predicted PKA targets in the cardiac myocyte, including some sarcomeric proteins, viz. cMyBPC, cTNI, ENO1, ENO3, CARP and COMMD4 (Tables 1 and 2). Moreover, we show that MMGL isoform 4 interacts with two regulatory subunits of PKA (Figure 3). Together these results describe MMGL isoform 4 as a novel sarcomeric AKAP, which, like mAKAP [14], is involved in assembling a PKA/PDE cAMP signalling module.

In addition to interacting with both types of regulatory subunits, viz. RI and RII, which qualifies MMGL isoform 4 as a dual-specific AKAP [11,19]. MMGL also meets the three other criteria for classification as an AKAP: By verifying its interaction with its proposed binding partners, we demonstrate that the targeting domains contained within the proposed AKAP participates in protein-protein interactions [11]. Further, it has previously been shown that MMGL is a phosphodiesterase 4D-interacting protein [13], thus, it meets the criterion for being able to coordinate multiple signalling pathways by anchoring additional signalling enzymes [11,20]. Lastly, we have shown in other Y2H screens that cMyBPC also binds to COMMD4 (unpublished results), here shown to be a MMGL interactor, while COMMD4 itself also binds to ENO1 and SNX3 (unpublished results). This strongly suggests that MMGL is part of a larger, multiprotein unit [11,20], and that MMGL isoform 4 may function as a vital link in signaling between upstream activators and numerous downstream targets [21].

Co-compartmentalization of both PKA and PDE4D is crucial for maintaining specificity of adrenergic signaling, and for maintaining contractility in cardiac cells [16]. We here established an important and novel link between PKA and PDE4D co-compartmentalization at the sarcomere level, and cMyBPC phosphorylation, and hence, regulation of cardiac contraction. The mechanism of docking of PKA to cMyBPC for phosphorylation of the MyBPC motif has previously not been elucidated; this study strongly suggests that MMGL isoform 4 anchors PKA to the N-terminal region, viz. C1-C2, of cMyBPC. The interaction between MMGL isoform 4, PKA, PDE4D and the N-terminal region of cMyBPC therefore sheds light on how second messenger responses are regulated in this particular part of the sarcomere.

We also found that β-adrenergic stimulation led to greater co-localization of myomegalin with both cMyBPC and cTNI in live cardiomyocytes, as evidenced by the increase in yellow staining during fluorescence microscopy in Figures 1 &5, respectively. Thus, although MMGL is apparently present within the sarcomere under normal conditions, this implies that under adrenergic stimulation, and consequential increased intracellular levels of cAMP, PKA is dynamically recruited by MMGL isoform 4 to distinct sarcomeric locations. This translocation of MMGL to the sarcomeric region is therefore compatible with a mechanism that would lead to increased phosphorylation of cMyBPC and cTNI, which is known to form part of the cardiac cellular stress response that leads to increased cardiac contraction [22]. Furthermore, given MMGL's known interaction with PDE4D [13], the mechanism for termination of the second messenger response, by degrading cAMP, would also be on site; lower levels of cAMP may then cause this multiprotein complex to dissociate again.

The knockdown studies of MMGL further suggests that MMGL not only acts as an AKAP at the MyBPC motif, but by implication plays a role in cardioprotection during adrenergic signaling. Although, in the presence of MMGL, all phosphorylation isoforms of cMyBPC are expressed well in H9C2 cells, and the level of the trisphosphorylated cMyBPC is increased in such cells under conditions of β-adrenergic stimulation as is to be expected, knockdown of MMGL under adrenergic conditions dramatically reduced cMyBPC expression (Figure 7). The latter finding suggests that when MMGL expression is reduced, cMyBPC phosphorylation is hindered, rendering the protein vulnerable to cleavage by proteases and reducing cMyBPC protein levels in the cell, as described by others [17,18,23]. Typically, annulment of the effect of an AKAP is commonly more noticeable on the target protein only after adrenergic stimulation: for instance, the studies of McConnell *et al*. (2009) [24] and Fink *et al. *(2001) [16] showed no significant difference in the level of phosphorylation of key cardiac proteins between wild-type cardiomyocytes and those in which AKAP function had been impaired with the Ht31 peptide. However, upon isoproterenol stimulation, dramatically decreased levels of PKA phosphorylation of all these proteins was observed in Ht31-transfected cells compared to isoproterenol-stimulated wild-type cells. Fink *et al*. (2001) [16] also demonstrated that AKAPs regulate phosphorylation of these PKA targets, including cTNI and cMyBPC, in response to β-adrenergic stimulation, although it has been unknown which AKAP/s are responsible for the phosphorylation of these two proteins up until our study. Thus, it appears that MMGL is an essential part in the β-adrenergic pathway leading to trisphosphorylation of cMyBPC and protection of the protein against degradation and normal sarcomeric integrity, which in turn is essential for normal physiological cardiac function, as well as cardioprotection during ischemia-reperfusion injury [25].

The subcellular localization and functions of some of the putative PKA targets identified via the Y2H library screen suggest that MMGL may act as AKAP in regions outside the sarcomere as well. Although some of the identified interactions, such as those with cMyBPC and cTNI, certainly occur within the sarcomere, associations with the other identified interactors (Table 2) do not necessarily occur within the sarcomere, nor do they necessarily all occur concurrently. In fact, a multiprotein subunit in the sarcomere consisting simultaneously of all identified MMGL-ligands would most likely sterically hinder crossbridge formation, and is therefore improbable. However, interaction of MMGL with proteins such as COMMD4, CARP, ENO1 and ENO3 may facilitate control of efficient PKA phosphorylation of these proteins; the protein-protein interactions and/or activation of these proteins may thus be regulated.

Although this study prioritized investigation of the interaction between the N-terminal of cMyBPC and MMGL, the other putative interactions of this region of cMyBPC should be further explored. The absence of cMyBPC as a prey in the MMGL Y2H library screen is most likely explained by the known absence of cDNAs representing the N-terminals of large proteins in oligo dT-primed libraries, while the absence of PRKAR1A and PRKAR2A may relate to the stringency of selection during heterologous bait-matings during this Y2H screen [26].

Interaction between myomegalin, PKA and cMyBPC or cTNI is also relevant to understanding of HCM patho-aetiology, as both of the latter proteins cause HCM when defective. It is known that point mutations in the C1-C2 region and in the MyBPC motif lead to HCM [3,27]; a potential mechanism for this may be involve disruption of binding between MMGL and cMyBPC. Such disruption would have consequences for PKA-phosphorylation of the cMyBPC motif (with implications for regulation of cardiac contractility), implying a poison-peptide mechanism underlying disease, as well as maintenance of sufficient cMyBPC levels within the sarcomere, particularly under conditions of adrenergic stress, implying a haplo-insufficiency mechanism. Point mutations in cTNI may have a similar patho-aetiology. It could be further speculated that, mutations in MMGL could similarly lead to inadequate binding of PKA and/or its sarcomeric partners, which might affect cMyBPC or cTNI phosphorylation and hence affect adaptation of cardiac contractility to adrenergic stimulation. Future studies could therefore consider MMGL as either a candidate causal gene or a potential modifier gene for HCM.

## Conclusions

This study shows that myomegalin isoform 4 is a novel sarcomeric AKAP, which forms part of a multiprotein complex that functions in cAMP signalling. It is particularly relevant to phosphorylation of cMyBPC and cTNI, and therefore, is of significance in the regulation of cardiac contractility in both health and disease.

## Methods

### Plasmid constructs

#### Y2H constructs

The region of MYBPC3 encoding domains C1-C2 was PCR-amplified from a MYBPC3 cDNA clone (kind gift of Prof Hugh Watkins, Oxford University). PCR-based site-directed mutagenesis, as previously described by Elliott *et al*. [28], was then used to generate a PCR fragment representing domains C1-C2 in which the codons for serines within all three phosphorylation sites of the cMyBPC motif were mutated to encode glutamic acids to mimic the trisphosphorylated state (PPP). The full-length cDNA from MMGL isoform 4 was amplified from a commercial construct, in vector pdEYFP-C1 (imaGenes GmbH). These fragments were individually cloned into the *Nde*I and *Eco*RI restriction sites in-frame with *GAL4*BD in the Y2H bait yeast expression vector pGBKT7 (Clontech) for use in the respective Y2H library screens or in Y2H-based direct protein-protein experiments.

The cDNA of the two PKA regulatory isoforms (*PRKAR1A *and *PRKAR2A*) were PCR amplified from a cardiac cDNA library (Clontech). These fragments were cloned into the *Bam*HI and *Xho*I restriction sites or the *Nco*I and *Bam*HI sites, respectively, of the Y2H prey vector pACT2 (Clontech).

Integrity of insert sequences, reading frame and cloning sites were verified by means of bi-directional sequencing, after which pGBKT7-PPP and pGBKT7-MMGL were transformed into *S. cerevisiae *strain AH109, and pACT2-R1A and pACT2-R2A into strain Y187 (Clontech).

#### Constructs used for verification analyses

The cDNAs from the putative interactors of MMGL isoform 4 identified in the Y2H library screen viz. *TNNI3, CARP, COMMD4, ENO1 *and, *ENO3*, as well as *PRKAR1A *and *PRKAR2A*, were PCR amplified and cloned into the pGFP2-C1 fluorescent vector (BD Bioscience). *MMGL *was further subcloned from the pGBKT7-MMGL construct into the pDs-Red-C1 vector (dsRed-MMGL) (BD Bioscience). The integrity of the cloning sites, reading frames and all interactor sequences were verified by bi-directional sequencing. These constructs were subsequently used in 3D *in vivo *co-localization and *in vivo *co-immunoprecipitation analyses.

### Y2H library screening

A *S. cerevisciae *Y187 pre-transformed human MATCHMAKER™ cardiac cDNA library constructed in pACT2 (BD Bioscience) was mated with the AH109 strain transformed with pGBKT7-PPP and the library screen performed according to manufacturer's instructions. Clones that expressed all three reporter genes, *HIS3, ADE2*, and *MEL1*, were further analyzed. An interaction-specificity test was used to identify preys that did not activate reporter genes in the presence of the following heterologous baits: pGBKT7-C5 (encoding Igl domain C5 of cMyBPC), pGBKT7-53 (encoding murine p53) and unrecombined pGBKT7. Prey plasmids interacting specifically with PPP were sequenced using a vector specific primer, and in-frame ORF sequences analyzed via BLASTN and BLASTP http://ncbi.nlm.nih.gov/blast to assign identity. Literature and public database searches were used to prioritize putative ligands according to function and subcellular localization. A Y2H library screen with pGBKT7-MMGL was subsequently similarly performed.

### Direct Y2H protein-protein interaction assay

pGBKT7-MMGL in yeast strain AH109 was mated individually with pACT2-R1A and pACT2-R2A in yeast strain Y187 to determine whether the PKA regulatory subunits interacts directly with MMGL. A single colony of pGBKT7-MMGL in AH109 was taken from appropriate selection plates and suspended with a single colony of pACT2-R1A or -R2A in YPDA media overnight. Mating mixes were plated onto solid growth medium lacking leucine (Leu) and tryptophan (Trp). Following an incubation period of four days, the colonies on each plate were transferred to medium lacking Leu, Trp and histidine (His), and incubated five days. Surviving colonies were finally transferred to medium lacking Leu, Trp, His and adenine (Ade) and growth was assessed on day 4 and 7. Control matings were included those of pGBKT7-MMGL with pACT2-cTNI(+), pACT2-TUSC4(-) and empty pACT2(-).

### Antibodies

Anti-HA, anti-Myc and anti-CARP antibodies were purchased from Santa Cruz Biotechnology. Living colours™ anti-dsRed (directed against dsRed-tagged proteins) and JL-8 (directed against GFP and YFP-tagged proteins) antibodies were purchased from Clontech and anti-β-actin antibody from Cell Signaling Technology. Anti-ENO1, -ENO3, -PRKAR1A, -PRKAR2A and -cTNI antibodies were purchased from Abnova.

### Cell culture and transfection

H9C2 cells were maintained at 37°C and 5% CO2 in Dulbecco's modified Eagle's medium, supplemented with 10% fetal bovine serum, 100 μg/ml penicillin and 100 μg/ml streptomycin. For *in vivo *co-immunoprecipitation, approximately 4 million H9C2 cells were seeded per 135 mm petri dish, and were transfected once 70-80% confluency was reached. Genejuice^® ^(Novagen) was used for transfections according to manufacturer's instructions. For 3D *in vivo *co-localization, approximately 20 000 - 30 000 H9C2 cells were seeded per well in 8-well borosilicate coverglass chambers (Nunc), and transfected after 70-80% confluency have been reached using Lipofectamine™ (Invitrogen) according to manufacturer's recommendations. Cells were differentiated into myotubes 24 h after transfection by changing the growth media to differentiation media (Dulbecco's modified Eagle's medium, 1% horse serum, 100 μg/ml penicillin and 100 μg/ml streptomycin). Fresh differentiation media was added every 48 h until cells were fully differentiated.

### Three-dimensional *in vivo *co-localization

Prior to image acquisition, the differentiation media was removed from the co-transfected, differentiated H9C2 cells and replaced with culture media containing a 1:200 dilution of Hoechst H-33342 nucleic acid stain (Sigma). Cells in three wells co-transfected with GFP-MyBPC/dsRed-MMGL were photographed using an Olympus IX 81 motorised inverted microscope (Olympus); after photography, cells were treated with 0.1 μM isoproterenol in order to stimulate phosphorylation of the MyBPC motif [7] and the same cells photographed again, to monitor changes in co-localization upon adrenergic stimulation. CellR software was used to perform image analysis. Z-stacks were done in order to co-localize the tagged proteins in three dimensions. Double-labelled images, utilizing the co-transfected samples, were obtained at different focal planes which were processed by the CellR software to determine co-localization. A 60X oil immersion objective was used to collect image stacks at 0.26 μm intervals in the plane. Subsequently, each co-localized image was created from the average of 25 frames. Change in co-localization was calculated using the CellR software and presented as a false colour image and percent co-localization as described by Loos *et al*., 2008 [29]. Changes in co-localization of MMGL isoform 4 and cTNI were obtained in a similar manner.

### *In vitro *protein transcription and translation

The cDNA encoding the native as well as the trisphosphorylated mimic of the C1-C2 region of cMyBPC and the full-length MMGL isoform 4 cDNA were PCR amplified to generate fragments that incorporates the T7 promoter and Myc- or HA-epitope tags, respectively. The TnT Quick Coupled Transcription/Translation System (Promega) was then used to transcribe and translate these PCR products into Met-35S-radiolabelled proteins, according to manufacturer's instructions.

### *In vitro *co-immunoprecipitation

Five microlitres of the respective 35S-labelled HA-MMGL and Myc-C1-C2 were mixed and incubated for 1 h at room temperature. One microlitre of anti-HA or anti-Myc antibody (5 μg/ml) was added followed by incubation of 1 h. Ten microlitres of pre-washed protein G agarose (Kirkegaard & Perry Laboratories) were subsequently added, together with 135 μl Co-IP buffer [5 mM phosphate-buffered saline (PBS), 5 μg/ml aprotinin, 0.5 mM PMSF, 100 mM DTT, 1% Tween-20]. Incubation of 1 h at 4°C followed on a rotating device. Centrifugation followed for 30 s at 3000 *g*, after which the pellet was washed five times with Tris-buffered saline with Tween-20 [20 mM Tris-HCl, pH7.5, 150 mM NaCl, 1% Tween-20]. Single immunoprecipitations were performed in the same way, using the same volumes of either bait or prey transcripts as were used in the co-immunoprecipitation reactions. Samples were electrophoresed on a 20% SDS-polyacrylamide gel, and protein bands were detected by autoradiography.

### *In vivo *co-immunoprecipitation

H9C2 cells transfected with the appropriate constructs were harvested 48 h following transfection, centrifuged at 3000 rpm for 3 min and the pellet washed with PBS. Two hundred microlitres of passive lysis buffer (PLB) [0.5 M EDTA, 1 M NaVO4, 10 mM Nappi, 1 M HEPES, 5 M NaCl, 1% TritonX, protease inhibitors and phenylmethylsulfonyl fluoride (PMSF)] was added to each sample. Samples were then incubated on ice for 30 min followed by centrifugation at 14000 rpm and the supernatants collected. Lysate concentration was determined via a Bradford assay, and the volumes equalized to 200 μl by adding PLB containing protease inhibitors and PMSF with a final concentration of 200 μg/μl. Lysates were pre-cleared by adding 20 μl Protein G agarose beads (KPL) and incubating samples at 4°C for 2 h on a rotating device. Fluorescent-tagged or endogenous proteins were immunoprecipitated from lysates using 1-2 μg of the appropriate antibody, as indicated in Figure 2c and 5. Following overnight incubation, 60 μl of protein G agarose beads was added to each sample, and incubated at 4°C for 2 h on a rotating device. Afterwards, immunoprecipitates were washed 5X in cold PLB containing protease inhibitors and PMSF, followed by resuspension in SDS loading buffer, and boiling prior to SDS-PAGE and subsequent Western blotting. Negative controls included samples in which protein G only was used in the absence of antibody, samples in which the HA-antibody was used as a negative control-antibody, and samples that had not been transfected with the dsRed-MMGL construct.

### RNA interference

The effect of siRNA transfection on myomegalin mRNA expression using different *PDE4DIP *siRNAs (Qiagen) was determined and optimized by a 2-step Q-RT-PCR using the Corbett Rotorgene system as follows: Approximately 4 × 10^4 ^H9C2 cells were seeded per well of an 8-well chamber slide, and siRNA transfected when cells reached 50-60% confluency, using HiPerFect transfection reagent (Qiagen) as per manufacturer's instructions. Total RNA extraction followed after 24 hours using the RNeasy Plus Mini kit (Qiagen) as per manufacturer's instructions. cDNA was subsequently transcribed using the Quantitect Reverse Transcription kit (Qiagen) as per manufacturer's instructions. The Quantifast SYBR green PCR kit (Qiagen) was then used to perform a real-time quantification of cDNA transcribed from the extracted RNA with or without non-silencing control (NSC) or PDE4DIP siRNAs. PDE4DIP levels were quantified with reference to three rodent reference genes (transferring receptor - *TRFR*, glyceraldehydes-3-phosphate dehydrogenase - *GAPDH *and heat shock protein 1β- *Hsp1β*) selected from a panel of 6 commonly used housekeeping genes.

The Genomewide designed non-validated Rn_RGD:708410_3_HP siRNA (Qiagen) resulted in the lowest MMGL gene expression quantification levels, and was used in subsequent experiments. Confluent H9C2 cells were transfected with GFP-tagged cMyBPC using Genejuice^® ^(Novagen). These cells were then transfected after 24 h with 10 nM *PDE4DIP *Rn_RGD:708410_3_HP siRNA using HiPerFect Transfection Reagent (Qiagen); control cells were not transfected with siRNA. For adrenergic stimulated cells, cells were treated with 65 mM CaCl2 for 10 min at 24 h post-transfection, followed by a 0.1 μM isoproterenol treatment for another 10 min. All cells were then lysed (as done for *in vivo *co-immunoprecipitation) and concentrations determined via Bradford assays, and all volumes equalized to 200 μl by adding PLB containing protease inhibitors and PMSF with a final concentration of 200 μg/μl. A non-denaturing immunoprecipitation of GFP-cMyBPC from the lysate followed using 1 μg of the JL-8 antibody with the Dynabeads^® ^Protein G and DynaMag™-2 system (Invitrogen) as per manufacturer's instructions. Isoelectric focusing of the GFP-cMyBPC immunoprecipitates followed to separate the four possible phosphorylation isoforms of cMyBPC.

### Isoelectric focusing

For the first dimension separation, the GFP-tagged cMyBPC immunoprecipitates were suspended in ReadyPrep 2-D Rehydration buffer 1 (Bio-Rad) containing Bio-lite pH 3-10 buffer (Bio-Rad) to a final volume of 200 μl. The protein/rehydration buffer mix was then applied to a pH 4-7 (11 cm) immobilized pH gradient (IPG) strip (Bio-Rad). Rehydration of the strip followed for 12 hours at room temperature. Afterwards, IEF was done under the following conditions: 8000 V for 20 min, 8000 V for 2 hours and 8000 V for 40 000 V hours at 21°C (Protean IEF Cell, Bio-Rad). Strips were stored at -80°C after IEF until required.

For 2-dimensional gel electrophoresis (2-DE), IPG strips were equilibrated by incubating the strips in equilibration buffer (0.375 M tris-HCl, 6 M urea, 20% glycerol, 2%, SDS) containing DTT (Sigma-Aldrich) for 15 min and then in equilibration buffer containing iodoacetamide (Sigma-Aldrich) for 15 min shaking at room temperature. Following equilibration, the IPG strips were placed on top of a Criterion XT 12% bis-tris precast gel (Bio-Rad) and sealed with 0.5% agarose (Bio-Rad). Electrophoresis of the second dimension separation was done under constant voltage (200 V) for 1-2 hours in 5% XT-MOPS running buffer (Bio-Rad). Gels were then transferred to a nitrocellulose membrane and subsequently western blotted with the JL-8 antibody.

## Authors' contributions

GMU carried out the MMGL Y2H direct protein-protein interactions and library screen, cell culture, 3D *in vivo *co-localization assays, *in vivo *co-immunoprecipitations, RNA interference and isoelectric focusing, and drafted the manuscript. AR carried out the cMyBPC-PPP Y2H library screen and *in vitro *co-immunoprecipitations. BL assisted with operating the Olympus IX 81 inverted microscope and CellR software. CJK assisted with interpretation of data and technical assistance. LJK and JM carried out cell culture and optimized siRNA transfections and MMGL knockdown. JR and JM assisted with siRNA and western blot optimization. JCM-S conceived of the study, acquired funding, participated in its design, coordination and interpretation of data, and revised and approved the final manuscript. All authors read and approved the final manuscript, and there are no conflicts of interest.
